# Uncovering the role of Symbiodiniaceae assemblage composition and abundance in coral bleaching response by minimizing sampling and evolutionary biases

**DOI:** 10.1186/s12866-020-01765-z

**Published:** 2020-05-19

**Authors:** Timothy D. Swain, Simon Lax, Vadim Backman, Luisa A. Marcelino

**Affiliations:** 1 Department of Civil and Environmental Engineering, Northwestern University, Evanston, IL 60208 USA; 2 Integrative Research Center, Field Museum of Natural History, Chicago, IL 60605 USA; 3 Department of Marine and Environmental Science, Nova Southeastern University, Dania Beach, FL 33004 USA; 4 Department of Ecology and Evolution, University of Chicago, Chicago, IL 60637 USA; 5 Physics of Living Systems, Department of Physics, Massachusetts Institute of Technology, Cambridge, MA 02139 USA; 6 Department of Biomedical Engineering, Northwestern University, Evanston, IL 60208 USA

**Keywords:** Climate change, Evenness, Phylogenetic comparative methods, Rarefaction, Richness, Symbiosis

## Abstract

**Background:**

Biodiversity and productivity of coral-reef ecosystems depend upon reef-building corals and their associations with endosymbiotic Symbiodiniaceae*,* which offer diverse functional capabilities to their hosts. The number of unique symbiotic partners (richness) and relative abundances (evenness) have been hypothesized to affect host response to climate change induced thermal stress. Symbiodiniaceae assemblages with many unique phylotypes may provide greater physiological flexibility or form less stable symbioses; assemblages with low abundance phylotypes may allow corals to retain thermotolerant symbionts or represent associations with less-suitable symbionts.

**Results:**

Here we demonstrate that true richness of Symbiodiniaceae phylotype assemblages is generally not discoverable from direct enumeration of unique phylotypes in association records and that cross host-species comparisons are biased by sampling and evolutionary patterns among species. These biases can be minimized through rarefaction of richness (rarefied-richness) and evenness (Probability of Interspecific Encounter, PIE), and analyses that account for phylogenetic patterns. These standardized metrics were calculated for individual Symbiodiniaceae assemblages composed of 377 unique *ITS2* phylotypes associated with 123 coral species. Rarefied-richness minimized correlations with sampling effort, while maintaining important underlying characteristics across host bathymetry and geography. Phylogenetic comparative methods reveal significant increases in coral bleaching and mortality associated with increasing Symbiodiniaceae assemblage richness and evenness at the level of host species.

**Conclusions:**

These results indicate that the potential flexibility afforded by assemblages characterized by many phylotypes present at similar relative abundances does not result in decreased bleaching risk and point to the need to characterize the overall functional and genetic diversity of Symbiodiniaceae assemblages to quantify their effect on host fitness under climate change.

## Background

Coral-Symbiodiniaceae symbioses are the foundation of coral reef ecosystems, as most shallow corals depend upon the photosynthesis of mutualistic Symbiodiniaceae for their fixed carbon [1]. When disassociated from these symbionts (e.g. bleaching), corals face decreased functionality and greater mortality which can result in systemic reef degradation and potential ecosystem collapse [2, 3]. Symbiodiniaceae (formerly known as *Symbiodinium* [4]) are genetically and physiologically diverse with > 400 phylotypes identified [5, 6] with different capabilities for photosynthetic production [7, 8], oxidative stress resistance [9], and thermal stress tolerance [10–13]. From this functionally diverse pool of potential partners, coral species associate with a selection of phylotypes (heretofore ‘Symbiodiniaceae assemblage’), where individual coral colonies frequently associate with a single symbiont phylotype, and across conspecific colonies few phylotypes are typically present at high frequencies and dominate the assemblage, while others are present at low frequencies and are minor components [14–16]. High-resolution genetic markers (e.g., nuclear microsatellites and chloroplast *psbA*^*ncr*^) are uncovering an unprecedented number of host-specific Symbiodiniaceae lineages revealing that ancestral phylotypes long considered generalists, such as *Cladocopium* C1 and C3 (*ITS2* type C1 and C3 sensu [17]) are likely subdivided in 100 s of host-specialized lineages with some flexibility in specificity, although a few lineages, such as *Durisdinium trenchii* (*ITS2* type D1–4 or D1a, sensu [17]) are host-generalists [4, 18–24]. Furthermore, these associations can be highly dynamic with seasonal changes in symbiont density [25–27], biogeographical changes in symbiont composition [14, 28, 29] or changes in phylotype composition due to environmental stress [29, 30]. Here, we focus on the ‘Symbiodiniaceae assemblage’ as the potential pool of symbionts available for association with a given host species across biogeographic distributions, environmental stress, and individual variability. The genetic and physiologic diversity of symbionts, host specificities, and dynamics of Symbiodiniaceae phylotype associations with each coral species have been challenging to adequately characterize, therefore their role in holobiont fitness and stress resistance is still being revealed. However, increasingly frequent and pervasive thermal stress under climate change has heightened the urgency to understand the consequences of coral-Symbiodiniaceae association patterns in response to thermal stress.

Associating with high thermotolerance phylotypes can raise bleaching thresholds by 1–2 °C and protect holobionts from thermal stress [10, 31, 32] by increasing the abundance of thermotolerant phylotypes within the assemblage (*symbiont shuffling*) or acquiring novel thermotolerant phylotypes from the environment (*symbiont switching*) (Adaptive Bleaching Hypothesis) [33–36]. This suggests that assemblage composition with greater phylotype richness (number of unique phylotypes associated with a coral species) may include phylotypes that provide functional redundancy or present the opportunity to dynamically adjust relative abundancies of phylotypes from different functional guilds to suit specific conditions, and thereby increase holobiont physiological flexibility [11, 37, 38]. Alternatively, high fidelity to fewer phylotypes may form associations that lack functional redundancy but result in matches of higher symbiont and host fitness that are more closely interdependent and robust and are therefore capable of thriving under diverse conditions [39, 40]. Recent analyses suggest that coral species with higher symbiont diversity may be more sensitive to environmental stress [30, 41].

The presence of sub-dominant (i.e., phylotypes present in the assemblage at low abundances) thermotolerant phylotypes in an assemblage permits shifting to be a viable strategy for thermal stress resistance. Phylotypes such as *Durusdinium trenchii* (D1–4) are known to increase host thermal stress resistance and are often present at background frequencies but increase in abundance under thermal stress as the previously dominant phylotypes decrease [11, 16, 42]. This suggests that assemblages that are characterized by uneven relative abundances of unique phylotypes (i.e. low assemblage evenness), may provide a mechanism of retaining functionally diverse phylotypes whose physiological capabilities are only needed under certain conditions. Alternatively, the presence of sub-dominant phylotypes may represent associations that have little functional significance [43] or are opportunistic non-mutualists [20, 44, 45] that destabilize the assemblage.

Here we revisit the hypothesized links between Symbiodiniaceae assemblage composition (richness) and relative phylotype abundance (evenness) with thermal stress resistance of the holobiont. As access to Symbiodiniaceae functional diversity is based upon phylotype genetic diversity, we reason that the physiological scope of Symbiodiniaceae assemblages can be assessed by characterizing phylotype richness and evenness using phylogenetic comparative methods to correct for evolutionary non-independence among species.

Species assemblage richness and evenness are foundational observations for assessing any ecological system, but several biases are known to affect their estimation [46–49]. Comparing raw species counts across assemblages can lead to misleading conclusions as the number of species has been observed to increase with increasing sampling effort [47, 49]. Species assemblage richness is typically evaluated by building rarefaction curves, which relate the expected number of species in each assemblage as a function of the number of samples [46]. Comparisons of raw species richness counts across assemblages are only credible when their rarefaction curves have reached an asymptote, such that raw-richness approximates true richness [47–49]. Compilations of Symbiodiniaceae assemblages from the literature include broad ranges of cumulative sampling effort and apparently diverse specificities of corals (e.g. [50, 51]), which will likely result in rarefaction curves of varying properties and of differing proximities to potential asymptotes. Additionally, richness assessments can be biased by species delineations in both Symbiodiniaceae and Scleractinia, which have been complicated by high genetic diversity (e.g., [4, 18, 52, 53]). The *internal transcribed spacer 2* (*ITS2*) of the ribosomal RNA nuclear gene is the most common taxonomic marker for Symbiodiniaceae, but its multiple copies per genome undergo concerted evolution resulting in a diversity of functional and non-functional copies (intragenomic diversity) that complicate species delineation [4, 18–20, 54]. Recent efforts involving high-resolution genetic markers (e.g., mitochondrial (*cob*), chloroplast (*psbA*^*ncr*^), and single copy nuclear microsatellites), together with genetic recombination, and physiological-, ecological- and morphological- differentiation, are clarifying definitions of genera, species, and individuals of both partners [4, 18–21, 52, 53, 55–57]. Furthermore, the ability to detect low-abundance phylotypes is variable, which may obscure our understanding of symbiont diversity as well as dynamic changes in partner identity or abundance. Molecular techniques, such as PCR coupled with Denaturing Gradient Gel Electrophoresis (DGGE) or direct sequencing have revealed dominant and co-dominant phylotypes (i.e., more than ~ 10% of the assemblage) which are assumed to be the most physiologically relevant and have shown dynamic changes in symbiont identity during thermal stress (e.g., [10, 58–60]). Recent high-resolution techniques such as quantitative PCR and next-generation sequencing (NGS) methods have uncovered low-abundance phylotypes (present at less than ~ 1% [15, 33, 42, 61, 62]) which, similarly to the ‘rare bacterial biosphere’, may provide functions necessary to the host and contribute to stress resilience [42, 62] or may be intragenomic variants (IGV) or represent individual variability within a lineage [18, 63, 64].

Additional biases occur in cross-species comparisons by ignoring the shared evolutionary history among species. Interaction patterns in mutualistic associations, such as pollinators or arthropods associated with ant hosts, are determined by a combination of ecological and evolutionary processes [65, 66]. Cross-species patterns of coral traits have been shown to be influenced by evolutionary relationships among species (e.g. coloniality and symbiosis, symbiont acquisition, skeletal light scattering properties, and partner specificity [67–70];). Therefore, it is likely that similar composition and relative abundancies of Symbiodiniaceae assemblages in corals may partially result from evolutionary processes [17, 18, 28, 30, 71]. Standard tests for phylogenetic structure in the data, and appropriate phylogenetic corrections for analyses that may be influenced by that structure, are widely applied to other groups [65, 72] and are becoming increasingly common as molecular phylogenetics of corals becomes more robust (e.g. [55]).

Here we determine the role of Symbiodiniaceae assemblage composition (richness) and phylotype relative abundance (evenness) in coral thermal stress resistance. We start by evaluating how sampling biases (insufficient and uneven sampling) affect the quantification of Symbiodiniaceae assemblage composition and abundance, and use rarefaction methods to standardize richness and evenness metrics to minimize biases. We then perform comparative analysis of their correlations with bleaching susceptibility across 123 coral species using phylogenetic comparative methods. We chose the *ITS2*-DGGE phylotypes for our analyses because it is the most extensive dataset currently available, with the understanding that (i) we are conservatively estimating richness as we are limited to the most abundant (marker sensitivity of ~ 10%) and likely most physiologically relevant phylotypes, and (ii) we will not have the best available resolving power to distinguish specific lineages or intragenomic variants identified by higher-resolution markers (e.g., [4, 19, 73]). We compiled and analyzed a dataset of 15,566 records of associations between 123 coral species with documented responses to thermal stress [74] and 377 Symbiodiniaceae *ITS2* phylotypes.

## Results

### Incorporating evolutionary history into cross-species analyses to reveal patterns among traits

We evaluated whether shared evolutionary history among coral hosts could explain similar coral-Symbiodiniaceae phylotype association patterns. Out of the fourteen regression analyses reported here, seven (50%) were corrected for phylogenetic relationships where significant phylogenetic signal was detected (Table S3).

### Effect of sample size on richness and evenness of Symbiodiniaceae assemblages

**Fig. 1:**
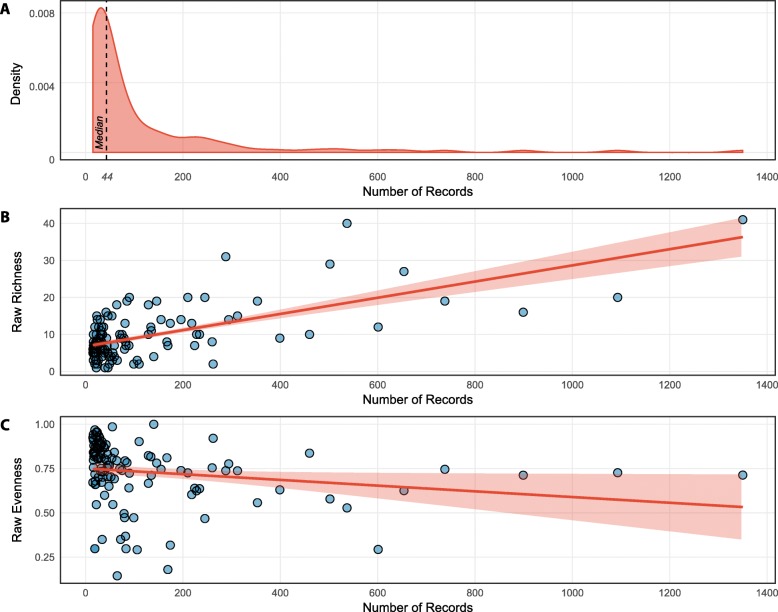
Sampling effort (number of coral phylotype association records) biases. **a** Probability distribution function of sampling effort across 123 coral species with 15 or more phylotype association records showing uneven sampling across species. **b** Raw-richness (maximum number of unique phylotypes within each Symbiodiniaceae assemblage) and **c** Raw-evenness (relative abundance of phylotypes within each Symbiodiniaceae assemblage) dependence on sampling effort for 123 species

We determined the species-specific number of association records to evaluate uniformity of sampling efforts across species (Table S1). The probability distribution function of the number of records of phylotype associations with each coral is a long-tailed distribution indicating that sampling effort is highly uneven, where half of the species have been sampled at 44 records of association or less (median = 44 records, *n* = 62 species), and few species have been extensively sampled (*n* = 22 species with > 200 records of association, Fig. 1a and Table S2).

We calculated raw-richness (raw-R) and raw-evenness (raw-E, Eq. 1) for each of the 123 coral-specific phylotype assemblages (Table S2), resulting in a mean raw-R of 9.55 ± 7 (mean ± st dev) phylotypes per assemblage and a range of 1–41 phylotypes, and a mean raw-E of 0.71 ± 0.21. Because the number of species observed is known to increase with sampling effort (e.g. [49]), raw-R and raw-E of phylotype assemblages were compared with sample size. Raw-R significantly increases with increasing sampling effort (linear-r = 0.656, *p* < 0.001, *n* = 123; Fig. 1b and Table S3), indicating that extensively sampled coral species may artificially appear to be associated with higher numbers of unique phylotypes and raw-richness may not reflect true richness of assemblages. Conversely, raw-E tended to decrease with sampling effort (linear-r = − 0.122, *p* = 0.179, *n* = 123; Fig. 1c and Table S3), as has been described for other assemblages (reviewed in [48]), but this trend was not statistically significant.

### Rarefaction analysis to compare phylotype richness across differentially sampled assemblages

**Fig. 2:**
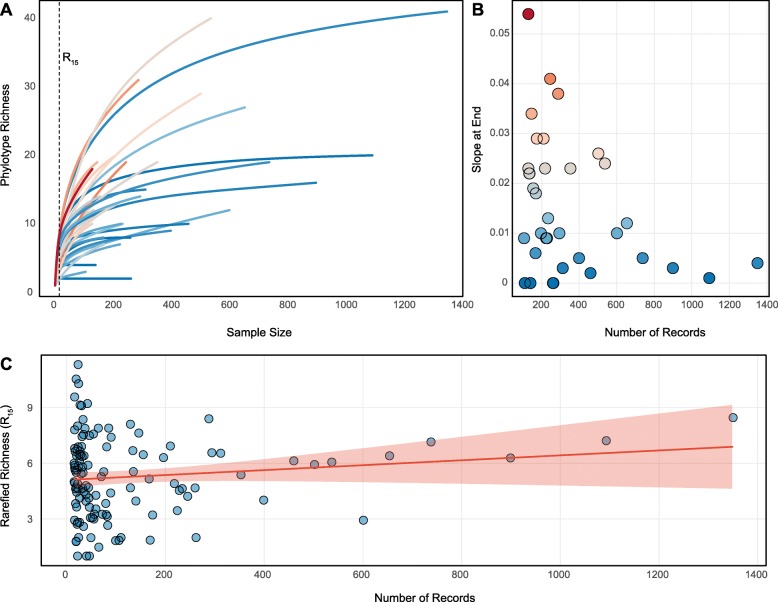
Rarefaction methods to estimate and compare phylotype richness among coral species. **a** Rarefaction curves for coral species with 100 or more records of coral-phylotype association (35 coral species, see Fig. S1 and Table S2 for details). Curves are colored by the extent to which they level off (‘slope at end’), with darker blue coloration indicating saturated curves (low slope at end) and darker red coloration indicating highly unsaturated curves (high slope at end) **b** Dependence of reaching an asymptote (expressed as slope at end of rarefaction curve) on sample size. Slopes approaching zero are indicative of asymptotic rarefaction curves. Coloration as in (**a**). **c** Dependence of rarefied-richness at rarefaction depth *n* = 15 (Eq. 2) on sample size (compare with Fig. 1b)

The tight correlation between sample size and raw-richness could be caused by insufficient sampling of the assemblage, which results in increased probabilities of detecting novel phylotypes with each additional sample. This effect is observable as a rarefaction curve (Eq. 2), which relates the expected number of phylotypes yielded by sampling effort. We focused on rarefaction curves for coral species with more than 100 records (35 species, Figs. 2a and S1, Table S2), as these assemblages are most likely to be sufficiently sampled. The slope at the end of the rarefaction curve (‘slope-at-end’) was calculated to evaluate if the curve reached an asymptote. As expected, the more an assemblage was sampled, the higher the likelihood a curve reached an asymptote (i.e. the smaller the ‘slope-at-end’, linear-r = − 0.338, *p* = 0.047, *n* = 35, Fig. 2B, and Table S3). Rarefaction curves of phylotype assemblages for a few coral species are asymptotic, indicating that raw-richness approximates true richness for those species (e.g. *Orbicella annularis* and *Pocillopora damicornis*, curve 7 and 1, Fig S1). However, many rarefaction curves are non-asymptotic with currently available sample sizes (e.g. *Porites lobata* and *Acropora millepora*, curves 2 and 21, Fig S1) and as in some microbial, invertebrate, and tropical plant assemblages they may never be asymptotic within practical sampling efforts ( [47] and references therein).

In order to compare phylotype richness across species with uneven and insufficient sampling, we used rarefaction to interpolate expected richness of each assemblage at uniform sample sizes (see methods, [48]). Specifically, we rarefied each curve to a standardized subsample size *n* (Eq. 2) selected in the rising part of the rarefaction curve and compared expected richness (rarefied-R) across assemblages. In this way, setting *n* = 15 records of association (R_15_ - only coral species with at least 15 records of association were analyzed, methods and Table S1) resulted in distinct rarefied-R_15_ values for Symbiodiniaceae assemblages indicated in Fig. 2a: 6.1 unique phylotypes associated with *Porites lobata*, 8.5 with *Pocillopora damicornis*, 7.2 with *Orbicella annularis* and 2.9 with *Acropora millepora* (see Table S2 and Fig S1 for rarefied-R_15_ calculated for other species).

**Fig. 3:**
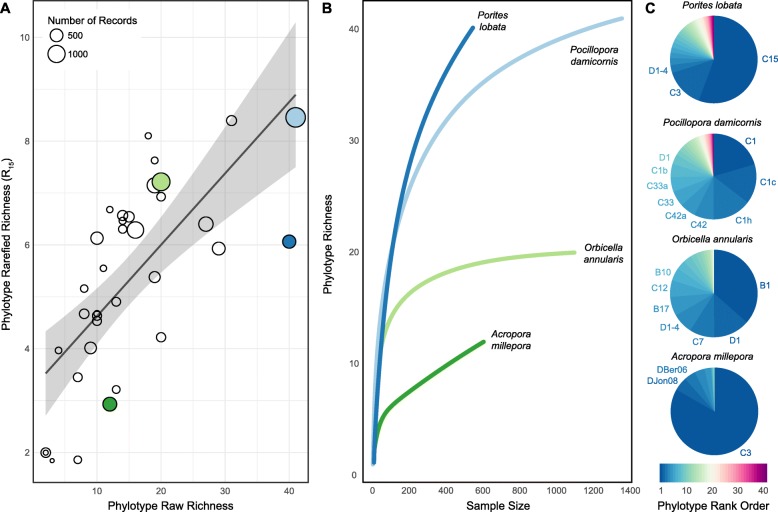
Comparing richness of Symbiodiniaceae assemblages across coral species. **a** Dependence of rarified- richness (at rarefaction depth *n* = 15, Eq. 2 or rarified-R_15_) on raw- richness for coral species with 100 or more records of coral-phylotype association (35 coral species, see Table S2 for details). **b** Rarefaction curves for exemplar species with rarified-R_15_ different from expected by the correlation (**c**)**.** Relative abundance of individual phylotypes in Symbiodiniaceae assemblages associated with the coral species shown in the rarefaction curves in (**b**). Phylotypes were ranked based on their individual abundances within each assemblage. Phylotypes present in the assemblage at frequencies of 5% or higher are labeled using their ITS2 nomenclature

We also determined if rarified-R_15_ was significantly affected by sampling effort. Contrary to the tight correlation between raw-R and sampling effort for 123 coral species (linear-r = 0.656, *p* < 0.001; Fig. 1b and Table S3), the relationship between rarefied-R_15_ and sampling effort is not significant and is nearly eliminated (linear-r = 0.127, *p* = 0.162; Fig. 2c and Table S3). These results indicate that better sampled phylotype assemblages do not have higher rarefied-R_15_ values than poorly sampled assemblages, allowing for valid cross-species comparisons of phylotype richness among assemblages that are insufficiently (non-asymptotic rarefaction curves) and unevenly sampled. Although rarefaction isolates rarefied-R from covariation with sampling effort, rarefied-R_15_ and raw-R are significantly correlated (phylo-r = 0.578, *p* < 0.001, *n* = 123 Fig. 3a, Table S3), indicating that underlying patterns in the data are preserved after rarefaction. Four exemplar species with varying rarefied-R_15_/raw-R correlations were examined: *Orbicella annularis* (rarefied-R_15_ = 7.2, raw-R = 20, raw-E = 0.73, and 1093 records) with rarefied-R_15_ higher than expected from the correlation, *Pocillopora damicornis* (rarefied-R_15_ = 8.5, raw-R = 41, raw-E = 0.71, and 1350 records) with rarefied-R_15_ within expected values, and *Porites lobata* (rarefied-R_15_ = 6.1, raw-R = 40, raw-E = 0.53 and 537 records) and *Acropora millepora* (rarefied-R_15_ = 2.9, raw-R = 12, raw-E = 0.29, and 601 records) with rarefied-R_15_ lower than expected from the correlation (Fig. 3a, Table S2). While *O. annularis* and *P. damicornis* have phylotype assemblages with asymptotic rarefaction curves, *P. lobata* and *A. millepora* have phylotype assemblages with non-asymptotic rarefaction curves (Fig. 3b). When the relative phylotype abundance of a Symbiodiniaceae assemblage is highly even (i.e. phylotypes are present in similar abundances) and the assemblage is highly sampled (e.g. *O. annularis*), it is likely that the assemblage is sufficiently sampled, as indicated by an asymptotic rarefaction curve, such that raw-richness approximates true richness (Fig. 3b and c). However, uneven relative phylotype abundance (i.e. presence of few high-abundance and multiple low-abundance phylotypes; low evenness) decreases the probability of sufficiently sampling and estimating true richness (e.g. *A. millepora*), particularly at smaller sample sizes (e.g. *P. lobata*) and may effectively prohibit asymptote identification at any sampling effort (e.g. *P. lobata*) (Fig. 3b and c).

### Rarefaction methods to compare phylotype evenness across coral species: rarefaction of slopes at start

Shape of rarefaction curves depends on the maximum number of phylotypes of the assemblage, which is defined by the asymptote (assemblage richness), and the relative abundance of species, which is defined by the slope (assemblage evenness) [48, 75]. Rarefaction curves of assemblages structured by high evenness have higher initial slopes in the rising part of the curve (i.e. ‘slope-at-start’), while assemblages structured by low evenness have lower initial slopes [48, 75]. This initial slope of the rarefaction curve is the maximum slope attainable and is mathematically equivalent to the probability of randomly selecting two different species from the compiled association records (PIE [76]). Probability of Interspecific Encounter ranges from 0, representing minimum evenness where one species is dominant and others are rare, to 1, representing maximum evenness where all species are equally abundant.

A similar dependence was observed for Symbiodiniaceae assemblages, where the greater the raw-E of the assemblage, the higher the PIE or the steeper the slope in the rising part of the rarefaction curve (linear-r = 0.855, *p* < 0.001, *n* = 123; Table S3). Specifically, rarefaction curves of low-evenness assemblages will slowly rise at first since each sample will likely yield a dominant phylotype before minor phylotypes are identified (i.e. low initial slope), while high-evenness assemblages will rise quickly at first since each sample will likely yield a novel phylotype (i.e. high initial slope, Fig. 3b and c). Similarly to raw-E (linear-r = − 1.22, *p* = 0.179, *n* = 123; Fig. 1c and Table S3), PIE is independent of sample size (phylo-r = 0.171, *p* = 0.058, *n* = 123, Table S3).

Symbiodiniaceae assemblages of the 123 coral species studied are structured by a broad range of relative phylotype abundance, such as high PIE of phylotype assemblages associated with *O. annularis* with several co-dominant phylotypes and low PIE of phylotype assemblages associated with *A. millepora* with one dominant and several sub-dominant types (Fig. 3c and Table S2). Sub-dominant phylotypes present in the assemblage at very low frequencies were observed only once (or a few times), so that for an assemblage sampled by 50 records ‘singletons’ would be present at 2%, while sampling 400 records would be 0.25%. Rarely observed phylotypes affect whether a rarefaction curve approaches an asymptote, such that assemblages with multiple rare phylotypes may need more extensive sampling to reach an asymptote or may never reach an asymptote at a practical sampling effort (compare *P. lobata* and *A. millepora*, with *O. annularis* and *P. damicornis*, Fig. 3c).

### Preservation of bathymetric and geographic patterns in phylotype assemblage richness

Comparing raw-R to rarefied-R indicates that underlying patterns in the data are preserved after rarefaction. However, there are other important characteristics of assemblage composition that could be potentially obscured during the rarefaction process. Studies of phylotype assemblages in coral species that span bathymetric and geographic ranges have shown significant correlations between phylotype richness and depth and biogeographic ranges of host coral species [28, 77–79]. We compared ranges of bathymetry, latitude, and longitude distribution for each coral species (Table S2) with the raw-R and rarefied-R of their Symbiodiniaceae assemblages (Table 1).

**Table 1:** Relationships between raw-richness (raw-R), rarefied-richness (rarefied-R), or taxon-specific bleaching response (taxon-BRI) and depth, latitude, or longitude ranges of observation records for 123 coral host species

Metric	Parameter	Linear ***r***	Linear ***p***	Pagel’s λ ***p***	Phylogenetic ***r***	Phylogenetic ***p***
**Raw-R**	**Species ∆ Depth**	0.145	0.110	**< 0.001**	0.24	**0.007**
	**Species ∆ Lat**	0.338	**< 0.001**	1.000	0.122	0.178
	**Species ∆ Long**	0.546	**< 0.001**	1.000	0.267	0.003
**Rarified-R**	**Species ∆ Depth**	0.003	0.978	**0.002**	0.220	**0.014**
	**Species ∆ Lat**	0.273	**0.002**	1.000	0.177	0.051
	**Species ∆ Long**	0.568	**< 0.001**	1.000	0.252	0.005
**Taxon-BRI**	**Species ∆ Depth**	0.110	0.224	**0.010**	0.112	0.217
	**Species ∆ Lat**	0.312	< 0.001	**0.043**	0.095	0.296
	**Species ∆ Long**	0.140	0.123	1.000	0.101	0.264

Significant Pagel’s λ *p*-values (< 0.05, in bold) indicate phylogenetic bias in the distribution of linear regression residuals; and only when Pagel’s λ is significant, were phylogenetically-corrected regression results accepted for interpretation. The *p*-values of regressions indicated by Pagel’s λ test (either linear for non-significant λ, or phylogenetic for significant λ) are also in bold when significant to indicate which result was accepted for interpretation. Individual species ranges of depth, latitude and longitude can be found in Table S2

**Fig. 4:**
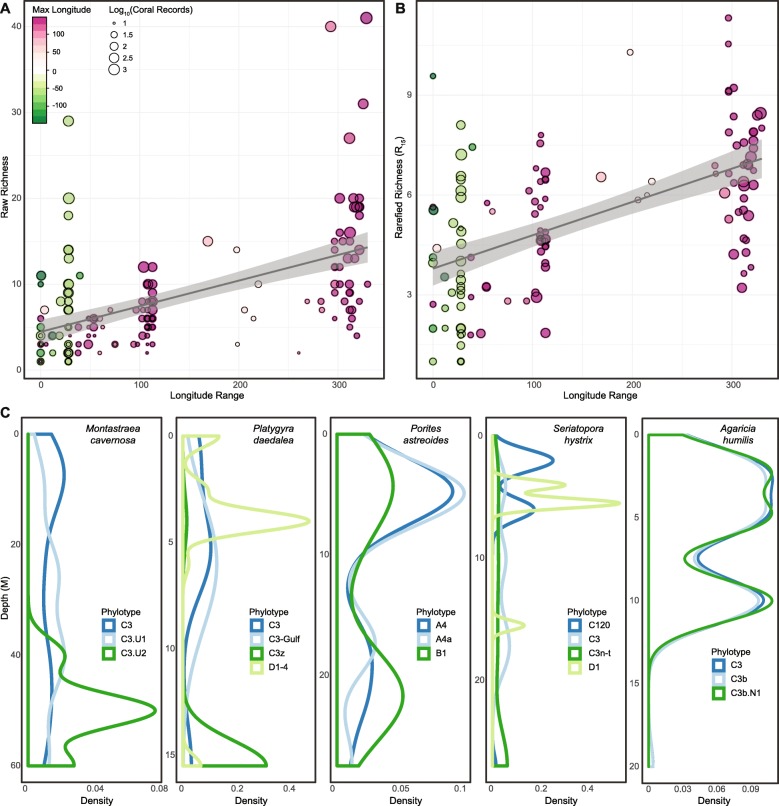
Differences in Symbiodiniaceae assemblage distribution with longitude and depth ranges. **a** Scatterplots of raw and rarefied-richness (at rarefaction depth *n* = 15) correlations with longitude range. **b** Probability distribution functions of individual Symbiodiniaceae phylotypes within an assemblage along different depth ranges (note that graphs have different maximal depth). Phylotypes which accounted for at least 10% of the records for each coral species were plotted as a function of depth to reveal changes in the frequency of association as depth increase. Each color corresponds to a phylotype, with the density of its records for that coral species plotted as a function of depth

Differences in distribution of coral species significantly correlated with both raw-R and rarefied-R, with wider range of bathymetry, latitude, and longitude correlated with greater phylotype richness (Table 1). These results indicate that rarefaction of phylotype richness preserves the general patterns of assemblage composition across host species distributions, with the strongest correlation observed across longitudinal range (expressed as ∆-longitude, linear-r = 0.568, *p* < 0.001, Table 1 and Fig. 4a). Across bathymetric ranges (expressed as ∆-depth, phylo-r = 0.220, *p* = 0.014, Table 1) some assemblages acquire new phylotypes more likely to be found at certain depths (e.g. phylotype C3.U2 in *Montastrea cavernosa* and phylotype D1–4 in *Platygyra daedalea*, Fig. 4b), while others mainly show differences in the relative abundance of some or all of their phylotypes (e.g. phylotypes A4 and B1 in *Porites astreoides*, phylotype D1 but not C3n-t in *Seriatopora hystrix*, and C3, C3b and C3b.N1 in *Agaricia humilis* Fig. 4b).

### Phylotype assemblage richness and evenness and their relationship to coral thermal stress response (coral bleaching susceptibility)

Using rarefied-R and PIE to minimize the effects of insufficient and uneven sampling and allow valid cross-species comparisons, we revisited the question of Symbiodiniaceae assemblage richness and evenness in expanding coral host functional capabilities under thermal stress.

**Fig. 5:**
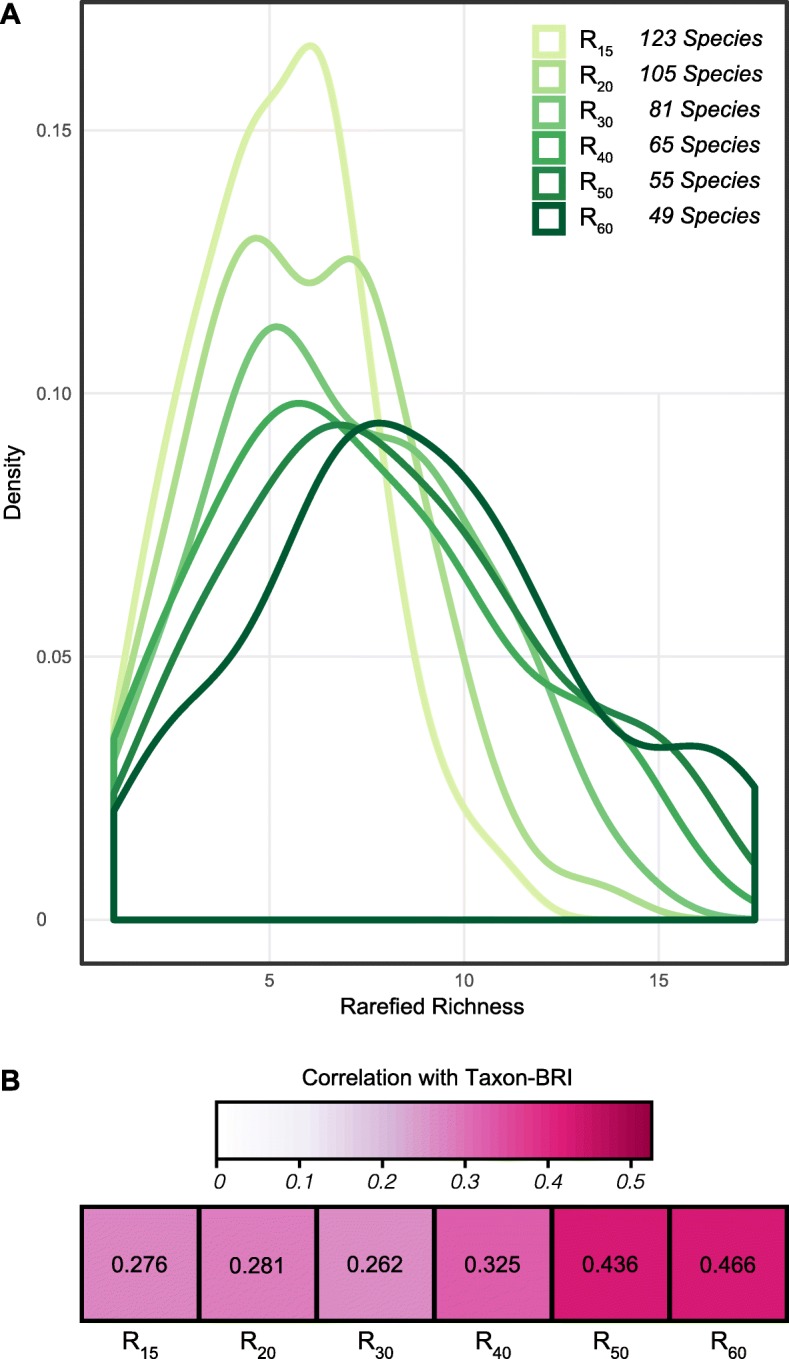
Rarefied-richness affects bleaching response (coral bleaching response index or taxon- BRI) in 123 coral species. **a** Probability distribution functions of rarefied richness at increasingly higher rarefaction depths with increasingly smaller species-sets. **b** Correlation between rarefied-richness and bleaching response for subsamples at increasingly higher rarefaction depths, indicating that corals associated with Symbiodiniaceae assemblages with higher number of phylotypes show an increased risk of coral bleaching. All correlations are significant (*p* < 0.02), see Table S3 for phylogenetic signal assessments

We evaluated the relationship between rarefied-R and coral bleaching response for 123 species (see Table S2 for species-specific susceptibility indices or taxon-BRI values) at multiple rarefaction depths. Performing correlations between richness and bleaching response at increasing rarefaction depths allows for evaluation of the trend and strength of correlation independently of differences in evenness among assemblages. Because assemblages with higher evenness show steeper slopes than assemblages with lower evenness (e.g. *P. lobata* and *A. millepora*, Fig. 3b and c), rarefaction curves of different assemblages may cross each other at some rarefaction depth depending on their expected richness. In this case, the rank order of expected richness between Symbiodiniaceae assemblages could change depending on the sub-sample size chosen for rarefaction. However, coral species lists that can be compared at increasing rarefaction depths will decrease in number because most coral species have small sample sizes (median = 20 records, Fig. 1a) and will be excluded from deeper analyses. Thus, rarefaction depths of 15, 20, 30, 40, 50, and 60 records yielded corresponding taxon sets of 123, 105, 81, 65, 55, and 49 coral species. We demonstrate that the probability distributions of rarefied-R shift with rarefaction depth, but converge at greater resampling depths (e.g. compare peak of distributions for rarefied-R_15_ ≈ 6.5 unique phylotypes out of a random draw of 15 records with rarefied-R_50_ ≈ 8 unique phylotypes out of random 60 record draws, Fig. 5a).

**Fig. 6:**
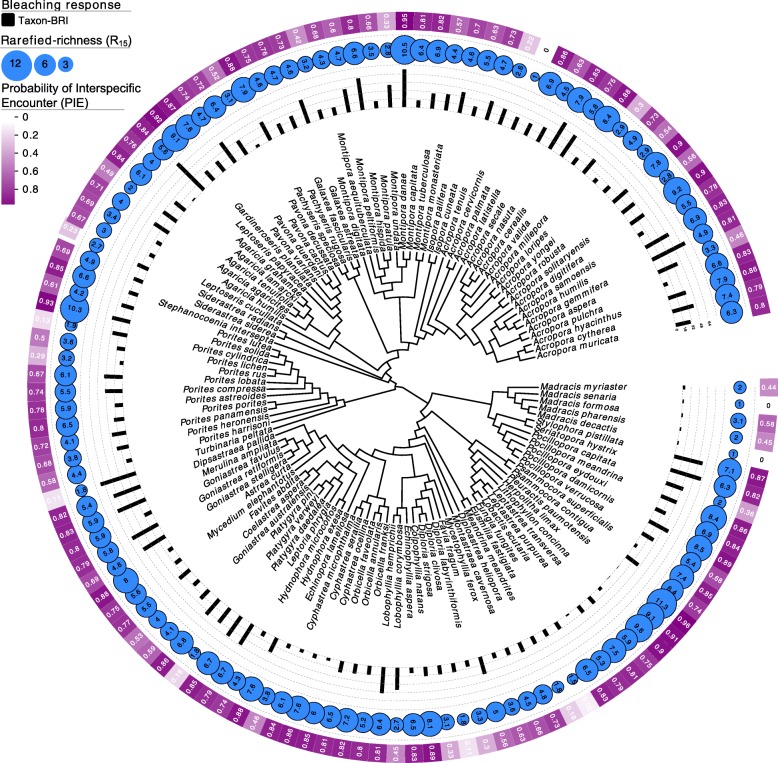
Coral phylogeny – mapping rarefied-richness (at rarefaction depth *n* = 15, Eq. 2, varying between 1 and 15), evenness (expressed as probability of interspecific encounter or PIE, Eq. 3, and varying between 0 and 1) and coral bleaching response index (taxon-BRI, varying between 0 and 100% [74]) on a phylogenetic tree of 123 species (see Table S2 for details)

The relationship between rarefied-R and bleaching susceptibility was examined at the same six rarefaction depths. We identified a significant positive correlation at all rarefaction depths, which increased in strength and significance with increasing rarefaction depth (correlation coefficients ranged from 0.276 at rarefaction depth 15, to 0.466 at rarefaction depth 60; Fig. 5b and 6 and Table S3). These results demonstrate that the relationship between taxon-BRI and richness is independent of rarefaction depth and its corresponding reductions in taxon sets. Furthermore, the relationship between taxon-BRI and richness is not being driven by increases in Symbiodiniaceae phylotype richness observed across host coral species distributions. Although we demonstrated a correlation between rarefied richness and coral host distributions (significant positive relationships between rarefied-R_15_ and ∆ depth, ∆ latitude, and ∆ longitude; Table 1), taxon-BRI is not significantly correlated with change in coral host ∆ depth (phylo-r = 0.112, *p* = 0.217, *n* = 123; Table 1), ∆ latitude (phylo-r = 0.095, *p* = 0.296, *n* = 123; Table 1), or ∆ longitude (linear-r = 0.14, *p* = 0.123, *n* = 123; Table 1). These results indicate that association with assemblages composed of increasing numbers of phylotypes (i.e. higher rarefied-R) is related to increasing risk of coral bleaching (i.e. higher taxon-BRI).

A similar pattern was detected between evenness and bleaching susceptibility, where a significant positive relationship between PIE and taxon-BRI was observed for 123 coral species (phylo-r = 0.182, *p* = 0.044, Fig. 6, Table S3). These results indicate that corals that associate with Symbiodiniaceae assemblages with equal relative abundance of phylotypes (high evenness) are more susceptible to bleaching.

## Discussion

Here we evaluated the effect of associating with different numbers of Symbiodiniaceae partners (assemblage composition or richness) at different abundances (assemblage evenness) on coral thermal stress response by performing comparative analysis across 123 coral species. Direct enumeration of species richness and evenness is known to be heavily biased by sample effort, and cross-species comparison is known to be heavily biased by evolutionary patterns among species. We minimized these biases by (i) applying rarefaction methods to derive standardized metrics of richness and evenness and (ii) applying phylogenetic comparative methods to correct for evolutionary relationships. We show that rarefaction methods effectively minimize the effects of sampling bias for cross-species comparisons (Figs. 1 and 2). Furthermore, the appropriate application of phylogenetic correction, or in the absence of phylogenetic signal the application of the uncorrected analysis, averted incorrectly identifying significance or lack thereof in five of the nine regression analyses (Table 1). Using these standardized metrics of richness and evenness along with phylogenetic comparative analysis, we show that coral species with higher Symbiodiniaceae assemblage richness and evenness are associated with a higher risk of bleaching.

Sampling effort of Symbiodiniaceae assemblages is highly uneven among 123 coral species in this study (Fig. 1a), in agreement with the previously observed highly skewed sampling effort among 307 coral species [80]. This is due, in part, to patterns of species abundance (few abundant species while most are rare [81, 82]), difficulty in sampling remote or deep reefs, and various biological or experimental interests of the investigators collecting the data (e.g. [28, 80, 83]). While enumerating unique phylotypes associated with each species (raw-richness or raw-R) and comparing across species has contributed to the understanding of coral specificity (e.g. [50, 84, 85]) it is unclear how much of the observed patterns of coral-Symbiodiniaceae associations are due to sampling biases caused by insufficient and unequal sampling effort.

We demonstrate that by rarefying each assemblage to a standardized subsample size, we can interpolate an estimate of expected richness for each assemblage (rarified-R) that is independent of variation in sample effort (Fig. 2a and c). Furthermore, because a standard subsample is chosen in the rising part of the rarefaction curve before an asymptote could be reached, rarified-R also becomes independent of the level of sampling required to reach an asymptote (Fig. 2a). Therefore, rarified-richness minimizes sampling effort bias and allows for estimates of expected phylotype richness regardless of how completely an assemblage is sampled (after achieving a minimum sampling effort) and allows for valid cross-assemblage comparisons. Rarefaction methods are broadly applied to obtain standardized metrics of richness to allow for comparisons among a variety of terrestrial, aquatic, and marine assemblages [47, 86].

Rarefaction of assemblage richness does not determine the absolute values of richness in the sample of coral-Symbiodiniaceae associations (i.e. number of unique phylotypes [87]), but instead calculates the expected number of unique phylotypes in a sub-sample randomly drawn from all association records with that coral species. The rarefaction process thus decouples the value of richness from sampling effort and provides a metric that can be validly compared across species while maintaining the underlying patterns in the data, including increasing richness with increasing bathymetric and geographic distributions of the host corals (Table 1, Fig. 4a). Exemplar Symbiodiniaceae assemblages that were tracked across bathymetry have greater richness values with expanding depth ranges, as phylotypes are substituted (*Montastrea cavernosa* or *Platygyra daedala*, Fig. 4b) or their relative frequencies are shuffled across depth (*Porites astreoides*, Fig. 4b). Similar changes in phylotype composition across environmental and biogeographic gradients have been reported in surveys of corals across the Great Barrier Reef (e.g., [88, 89]), Indian Ocean [28], Caribbean [83], in Red Sea *Porites* [90] and corals from the South China sea [91].

The relative phylotype abundance, or evenness, of Symbiodiniaceae assemblages was also evaluated. Raw-E varied considerably among the 123 Symbiodiniaceae assemblages in this study (range 0–1) and, although it tended to decrease with sample size (as observed for other communities [48]), this trend was not statistically significant (Fig. 1c). However, because rarefaction curves provide knowledge of both richness (by rarefying assemblages to a given rarefaction depth) and evenness (by determining the maximum slope of the rising part of the rarefaction curve; reviewed in [48]) we extracted both richness and evenness metrics directly from rarefaction curves for consistency. There is a strong correlation between PIE and raw-E for the 123 coral species (linear-r = 0.855, *p* < 0.001, Table S3), and because PIE is also decoupled from sampling effort, it can be validly compared across species.

We observed a strongly positive correlation between rarefied-richness and bleaching susceptibility, regardless of rarefaction depth (Fig. 5b). This correlation is not driven by patterns in host distributions (∆ depth, ∆ latitude, and ∆ longitude), which are significantly correlated with rarefied-R_15_, but not taxon-BRI. This indicates that increasing numbers of phylotypes in Symbiodiniaceae assemblages are associated with increasing coral susceptibility to bleaching. Our study extends the observations of Putnam et al. [30] for raw-richness in *Acropora*, *Pocillipora*, and *Porites* genera and for diversity in a larger sampling of coral species by Darling et al. [41], which reported increased sensitivity to environmental stress with increasing numbers of symbiont partners. We also observed a positive correlation between Symbiodiniaceae assemblage PIE and coral bleaching susceptibility (phylo-r = 0.182, *p* = 0.044, Table S3), where corals associated with assemblages dominated by one phylotype (PIE = 0, or → 0) showed lower bleaching susceptibility than corals with multiple co-dominant phylotypes (PIE = 1). This indicates that the presence of a dominant phylotype is associated with decreasing coral susceptibility to bleaching. Highly specific mutualisms (i.e., PIE = 0) may promote tight correlations between symbiont and host fitness [39], where symbiont fitness is associated with host survival, reproduction, and increased physiological performance of the holobiont under both normal or stressful conditions (eg., [19, 68, 92]). Alternatively, associations with one main phylotype and multiple sub-dominant phylotypes present at low-abundances (PIE → 0), may favor holobiont fitness under stress since sub-dominant phylotypes may represent a pool of diverse functional capabilities that can be accessed by the host as needed to address changes in environmental conditions and may temporarily replace dominant phylotypes [11, 15, 33]. Furthermore, associations with multiple phylotypes (PIE → 1) where hosts may expel symbionts for novel genotypes, (i.e. high symbiont turnover) may weaken the relationship between symbiont fitness and host survival. In this case, symbiont fitness may be ascribable to the relative importance of other determinants of symbiont performance [93] such as the ability to outcompete other symbionts (e.g. [94]) or to avoid host expulsion (e.g. [11]). When symbionts face these competing selection pressures, selection for increased holobiont fitness may be greatly reduced in favor of symbiont-selfish traits that may be detrimental for symbiosis stability under stress (e.g. [37, 93]). To elucidate the dynamics and the roles of both low-abundance and resident dominant phylotypes in the assemblages, high-sensitivity tools such as NGS may be required. Less sensitive DGGE-*ITS2* may not detect low abundance phylotypes in some surveys unless the resident dominant assemblage is disturbed by stresses. These hypotheses will become more easily testable as more studies using high resolution techniques examine the identities and relative abundances of Symbiodiniaceae associations at the colony-level and are reported on platforms like SymPortal [95].

### Limitations and general considerations

By identifying strong correlations between the number of symbiont partners, their relative abundance, and coral response to thermal stress, this study adds to the growing evidence that the fitness of the host is influenced by the overall functional capabilities of its Symbiodiniaceae assemblage, including dominant and sub-dominant phylotypes. Due to the large number of coral species used for comparative phylogenetic analysis, we applied the largest and most comprehensive currently available dataset: the ribosomal RNA *ITS2* Symbiodiniaceae phylotypes. Although *ITS2* is a multiple-copy gene with considerable intragenomic variation (reviewed in [54, 64, 96, 97]), it is the most intensively used genetic marker in Symbiodiniaceae research and is therefore comparable across studies and species, and is the most comprehensive source of information on our current understanding of Symbiodiniaceae differential host specificity, nutrient production, nutrient acquisition, photophysiology, and thermotolerance. However, our results should be interpreted within the limitations of *ITS2* phylotype identification.

First, we are limited to evaluating the most abundant (and likely most physiologically relevant) phylotypes present in each symbiont assemblage due to *ITS2*-DGGE sensitivity limits (~ 10%). Importantly, individual phylotype abundance seems to be dynamically regulated by the host in response to environmental change and stress [25, 98]. High-sensitivity techniques such as NGS are poised to advance our understanding of the diversity and dynamics of Symbiodiniaceae assemblages due to their ability to identify extremely low-abundance sequences (e.g., [61, 63, 64]). However, NGS tends to uncover very rare community members within a colony (less than ~ 1%) that have, so far, an unknown bearing on coral fitness (e.g., [42, 43, 61, 62]). These low abundance phylotypes could significantly influence rarefaction analysis by inflating richness estimates. Furthermore, because NGS does not yet have host species coverage provided by ITS2-DGGE (but see recent advances in [95]) and is very sensitive, the two data types are not easily merged, so NGS data was not included in this study.

Additionally, *ITS2*-DGGE has shown low taxonomic resolution to distinguish many recently identified host-specific lineages or IGVs identified by higher-resolution markers (e.g., [4, 19, 73]). Recent integrative approaches that rely upon multiple lines of evidence are supporting robust species hypotheses and clarifying species delineations in Symbiodiniaceae [18–21, 57]. Likewise, host misidentification, particularly in the case of cryptic coral species complexes, may also affect richness and evenness estimates since distinct species within the complex may associate with different phylotype assemblages. Furthermore, while there are many phylotypes that are typically identified by 2–3 co-dominate unique *ITS2* sequences (such as D1–4 or D1–4-6) and are widely accepted to represent a single *ITS2* phylotype because of repeated observation, there are other observations where it is much less clear what this variation may represent, such as C3.N5 or C3.N6 (of [78]) which were originally thought to represent IGV within single phylotypes or a mix of host-specific phylotypes, but recent evidence suggests the latter (Pim Bongaerts personal communication). These observations of similar *ITS2* DNA sequences that have not yet been codified in the phylotype literature were classified as potential intragenomic variants (P-IGVs, but not the unique variants described in Bongaerts and colleagues, see Table S1), which were treated as individual phylotypes in the analysis. P-IGVs make up ~ 4% of our phylotype association records (Table S1), and exclusion of these records (i.e., treating them as true IGV of a single phylotype) still show a significantly positive correlation between rarified-richness and BRI (phylo-r = 0.213, *p* = 0.02, *n* = 118) similar to the full dataset.

Finally, there are technical limitations regarding *ITS2*-DGGE identification that could confound estimates of symbiont richness and evenness. Our dataset contains *ITS2* phylotypes identified using different primers and protocols, although 67% of coral-Symbiodiniaceae association records (10,496 out of 15,566, Figure S2) were identified with ITSintfor2 and ITS2Clamp primers and protocol [17, 99] (Table S1). Recently, three of the most commonly used *ITS2* primers were tested and a specific pair was demonstrated to be the most specific and sensitive [100], rendering other primers less optimal to identify low-abundance phylotypes across a variety of host species.

Additionally, about 15% of the association records were identified using cloning of rDNA genes (Table S1), which is known to increase the variability of the sequences generated and artificially increase richness estimates [101]; nevertheless, because many of the phylotypes identified by cloning were common to the main phylotypes identified by *ITS2*-DGGE and artifacts are typically rare events, we expect them to have a negligible influence on the conclusions of this study. Furthermore, while most DGGE bands were excised, sequenced, and reported as containing unique phylotypes, or particular configurations of phylotypes if co-dominant bands were present (Table S1), IGVs may artificially overestimate richness in particular assemblages or may not be resolved by DGGE and underestimate richness [73]. However, in spite of these drawbacks, *ITS2*-DGGE has identified many phylotypes over the last decades that have proven congruent with higher-resolution genetic markers (e.g., [18]) and several compilations of coral-Symbiodiniaceae associations (e.g., [5, 102, 103], which are included in our dataset) have been analyzed to inform general trends and patterns.

High-resolution techniques that are providing genetic and metabolic characterization of all the members of Symbiodiniaceae assemblages (e.g., [42, 61, 62, 104]) will reveal a clearer picture of the functional capabilities that sub-dominant phylotypes may bring to their hosts, as well as the role of dominant and subdominant phylotypes in the community, as well as community dynamics. These colony-level data on Symbiodiniaceae assemblage composition, abundance, and dynamics may aid in management and conservation of coral species in a changing climate.

## Conclusions

We have demonstrated that rarefaction and phylogenetic comparative methods allows for the elimination of specific biases in assessing the relationship between richness and evenness of Symbiodiniaceae phylotype assemblages and coral bleaching response, while preserving underlying assemblage patterns across host bathymetry and geography. Although these techniques are broadly applied across many other systems, they are rarely applied to the coral-Symbiodiniaceae symbioses and, to our knowledge, estimates of these assemblages have never been simultaneously corrected for both sampling effort and phylogenetic biases. The results indicate that Symbiodiniaceae phylotype assemblages that are characterized by the potential flexibility afforded by many phylotypes present at similar relative abundances are associated with increased bleaching susceptibility across a broad scope of coral species diversity and suggest that coral species that employ this strategy in compiling their symbiotic assemblages will not be the species that persevere under climate change.

## Methods

### Pan-tropical matrix of associations between 123 coral species and 377 Symbiodiniaceae ITS2 phylotypes

Naturally occurring combinations of coral species and Symbiodiniaceae *ITS2* phylotypes were identified through a survey of the literature. We targeted coral species with quantifiable responses to thermal stress through a previously described taxon-specific bleaching and mortality index, taxon-BRI, where elevated response to thermal stress (high taxon-BRI) indicates high bleaching susceptibility [74]. However, only phylotype associations observed under non-bleaching conditions were considered here in order to identify the baseline pattern of Symbiodiniaceae associations for each coral species. Thus, taxon-BRI is used in this study as an estimate of the mean bleaching risk of each coral species due to the lack of information regarding bleaching response of each sampled colony. We relied upon Symbiodiniaceae phylotype definitions based on *ITS2*, without assuming that this level of genetic variation represents a specific taxonomic unit, but rather as an ecologically relevant unit of genetic diversity. *ITS2* phylotypes in our compiled dataset were typically identified through PCR amplification of the nuclear rDNA, combined with DGGE, band excision, and subsequent DNA sequencing (~ 75% of the records of association; ~ 15%, ~ 7.5% and ~ 0.9% were identified through cloning, single strand conformation polymorphism and small-subunit restriction fragment length polymorphism respectively). Table S1 contains detailed information on the technique described in each study used for phylotype identification. Individual phylotypes with repeatedly observed co-dominant DGGE bands were listed as different configurations; by a single *ITS2* DNA sequence (e.g. D1), two distinct sequences (e.g. D1–4), or three distinct sequences (e.g. D1–4–6) as reviewed in [21].

Symbiodiniaceae recently underwent major taxonomic revision with genetic groups known as clades (within formaly known *Symbiodinium* genus) now being recognized as distinct genera within the Symbiodiniaceae family [4]. We treat sets of sequences that are repeatedly identified together throughout the literature, and are broadly considered as part of the DGGE fingerprint of a single phylotype, as single analytical units (i.e. one set of sequences equals one phylotype) and list them by their new genus name and their earlier *ITS2* alphanumerical designation (Table S1). Observations of similar *ITS2* DNA sequences that are rare, singleton observations, or singleton reports that have not yet been codified (i.e., designated as an *ITS2* phylotype using the standard nomenclature) in the literature as being a component of a repeatedly observed and legitimate phylotype were classified as potential intragenomic variants (P-IGV) which were treated as individual phylotypes in the analysis (~ 4% of the association records, Table S1).

Existing association compilations [5, 102, 103, 105] and an original search of GenBank (www.ncbi.nlm.nih.gov/nucleotide/) and the scientific literature identified primary sources for the original observations of coral species-Symbiodiniaceae *ITS2* phylotype associations. Each recorded association in our dataset is one observation of a single Symbiodiniaceae phylotype in association with an individual coral colony and does not include repeated sampling of the same phylotype within the same coral colony. Different phylotypes co-occurring in the same coral colony are unique association observations. In order to minimize artifacts associated with scarce sampling, only coral species with at least 15 records of association were included in the compilation, yielding 15,566 records of associations between 123 coral species and 377 Symbiodiniaceae phylotypes (Table S1). Furthermore, the studies showed high congruency among protocols and *ITS2* primers used for phylotype identification, where in our dataset 10,496 out of 15,566 records (67% of records, Figure S2) were identified with ITSintfor2 and ITS2Clamp primers following the protocol originally described by LaJeunesse and colleagues [17, 99] (Table S1). Association records included information on the depth and location of sample collection, such that bathymetric, latitudinal, and longitudinal ranges could be calculated for each coral species (Table S2).

### Raw-richness and raw-evenness

Richness is a basic species (in Symbiodiniaceae, phylotype) composition parameter of any assemblage and provides the number of unique species within that assemblage but ignores the relative abundance of individual species by treating composition as a binary question of presence or absence. Here, raw-richness is the number of different phylotypes associated with a coral species in the dataset. Evenness describes the abundance structure of the assemblage by quantifying the uniformity of relative abundances of individual species in the assemblage. Thus, the number of interactions which are possible is quantified by richness, while how equally those interactions are realized is quantified by evenness. Raw evenness (*J’*) is estimated as:

1 $$ {J}^{\prime }=\frac{-{\sum}_{i=1}^P{p}_i\ln {p}_i}{\ln P} $$

where, $-{\sum}_{i=1}^P{p}_i\ln {p}_i$ is the Shannon diversity index of the set of associations with each coral, *p*_i_ is the proportion of all associations for the coral species that are with phylotype *i*, and *P* is the number of phylotypes that associate with that species (raw-richness).

### Rarefaction curve construction and insufficient sampling

A rarefaction curve represents expected richness in an assemblage as sub-sample size increases, which at infinitely large sample sizes hypothetically approaches true richness estimated as an asymptote [47, 106]. Records of Symbiodiniaceae phylotype associations were used to construct individual rarefaction curves for each coral species targeted. Rarefaction is calculated as:

2 $$ E(S)={\sum}_{i=1}^P\left\{1-\left[\left(\frac{N-{N}_i}{n}\right)/\left(\frac{N}{n}\right)\right]\right\} $$

where *E(S)* is the expected number of phylotypes in the rarefied coral sample, *n* is the standardized sample size, *N* is the total number of association records for the coral species, *N*_*i*_ is the number of association records for phylotype *i* with the coral species, and *P* is the number of unique phylotypes that associate with the coral species, or raw-richness. Thus, rarefaction calculates the expected number of phylotypes in a subsample of size *n*, which is randomly drawn from a pool of records *N* (in our case all phylotype records associated with a coral species). Rarefaction curves can be generated for every value of *n* in *N*, resulting in a smooth rarefaction curve that can be used to infer sufficient sampling, as the slope converges to 0 [47]. Rarefaction curves were calculated using *rarecurve* in the ‘Vegan’ R package v2.4–6 [107]. If the rarefaction curve of a coral species reaches an identifiable asymptote (its slope approaches 0), this was accepted as evidence of sufficient sampling, such that further sampling is not likely to significantly alter the curve nor the parameters that can be derived from it. To evaluate the extent to which each curve reached an asymptote, the ‘slope-at-end’ was calculated as the difference between rarefaction at depth *n* = *N* and rarefaction at depth *n* = N-1 (i.e. R_N_ – R_N-1_).

### Standardization of assemblage composition and abundance parameters

*Rarefied- richness*: Calculations of raw-richness values from assemblages with asymptotic rarefaction curves are valid for cross-species comparisons as raw values will approximate true values of the assemblage, although they should be interpreted as representing their respective minimum values [47]. However, calculations of raw-richness values from assemblages with non-asymptotic rarefaction curves are inappropriate to compare across species, since additional sampling will uncover further species [47, 75] rendering the comparisons inaccurate and potentially misleading. In this case, rarefaction methods can be applied to interpolate richness values from the rarefaction curves by setting a constant subsample *n* for each species in the rising part of the curve (rarefied-richness [47, 48];). For example, setting *n* to 15 association records (R_15_) on the x-axis (or rarefying each assemblage to *n* = 15) allowed for comparison of expected rarefied-richness across multiple assemblages by comparing values of the y-axis at a fixed *n* = 15. Thus, taking rarified-richness at a given rarefaction depth (subsample size *n*), is equivalent to randomly drawing a number of associations from the dataset equal to the rarefaction depth (without replacement) and finding a mean number of unique phylotypes detected across many iterations of these random draws. We calculated rarified-R at rarefaction depths of 15, 20, 30, 40, 50, and 60 records.

*Probability of Interspecific Encounter (or assemblage evenness):* the shape of rarefaction curves depends, in part, on the relative abundance of species in the assemblage, such that rarefaction curves from assemblages with co-dominant species (high evenness) will rise faster than those from assemblages with a mixture of dominant and rare species (low evenness) [48, 75]. Thus, the slope of the rising part of the rarefaction curve (maximum slope) can be used as the expectation of observing additional phylotypes resulting in a metric that is independent of sample size [48, 76]. Taking evenness as the maximum slope of the rarefaction curve is equivalent to Hurlberťs [76] Probability of Interspecific Encounter (PIE) defined as:

3 $$ PIE={\Delta }_1={\sum}_{i=1}^P\left(\frac{N_i}{N}\right)\ \left(\frac{N-{N}_i}{N-1}\right) $$

where *P* (as in Eq. 1) is the number of unique phylotypes that associate with coral species (raw-richness), *N* is the total number of records from the assemblage, *N*_*i*_ is the number of records of the *i*th phylotype in the assemblage. Probability of Interspecific Encounter is an alternative measure of evenness which defines the probability that two randomly chosen samples from an assemblage will be different phylotypes. Probability of Interspecific Encounter is equivalent to the difference between rarefaction at *n* = 1 and rarefaction at *n* = 2 (i.e. R_2_-R_*1*_) or the steepest slope in the rarefaction curve and is thus mathematically equivalent to rarified-evenness (reviewed in [48]).

### Phylogenetic correction

Species trait data often vary in a pattern that reflects their evolutionary history, where closely related species have more similar traits then distantly related species [65, 66]. This phylogenetic structuring of species trait data may also be present between pairs of species traits, where the relationship between two traits may covary with the phylogenetic distance between species. Phylogenetic structure in the relationship between species traits is detectable as phylogenetic signal in regression residuals; i.e. the deviation of each species trait value from the value predicted by the regression is significantly different from random and is significantly similar to a pattern described by the species phylogeny.

Significant phylogenetic signal in regression residuals can confound standard statistical analyses and artificially obscure significant patterns (reviewed in [108]). Standard statistical analyses do not properly account for information loss caused by similarities among evolutionary relatives, violating the assumption of replicate independence, and result in increased type I error rates (probability of incorrectly identifying statistical significance [109]). However, inappropriately applying phylogenetic correction to analyses of data that are not phylogenetically structured can result in poor statistical performance and uncorrected regression will return a more robust result [72]. Therefore, phylogenetically corrected analyses should be favored over standard statistical analysis only when the assumption of replicate independence is violated by demonstrable phylogenetic structure in the data.

The coral phylogeny of Huang [55] was trimmed to 123 targeted taxa using Phytools ver 0.6–20 R package [110] to maintain accurate relationships and branch lengths. This trimmed phylogeny was used to define evolutionary relationships and distances between species for detection of phylogenetic structure in the data and phylogenetic correction of regression analyses. A priori assessment of phylogenetic signal was performed on the residuals of Ordinary Least Squares (OLS) regression of each trait comparison analysis (see Table S3 for regression analysis between specific traits), which were then mapped to the phylogeny of corals (each residual value was assigned to its species in the data matrix), and tested for statistically significant phylogenetic signal (a pattern in the regression residual values that reflect phylogeny topology and branch lengths) with Pagel’s Lambda [111] using PhyloSig [112] in the Phytools [110] R package. Significant phylogenetic signal detected in the OLS residuals (i.e. Pagel’s λ, *p* < 0.05) indicates that phylogenetic correction of the regression analysis is warranted and that the OLS regression is biased by evolutionary relationships among species. Phylogenetic regression analysis was conducted via Phylogenetic Independent Contrasts (PIC) test applied to the same coral phylogeny and was performed in the Phenotypic Diversity Analysis Programs (PDAP:PDTREE [113];) module of Mesquite ver 3.10. Here we report the results of both the OLS and PIC analyses but use the significance of Pagel’s λ to determine which to interpret.

## Supplementary information

**Additional file 1: Table S1.** Dataset of interaction between 123 coral species and 377 phylotypes (15,566 records) and their associated biogeographic information. Symbiodiniaceae phylotypes are listed by their new genus name [4] and their earlier ITS2 alphanumerical designation. Methodological details such as technical approaches and primers used are listed per publication, and additional notes on the protocols are provided.

**Additional file 2: Table S2**. Species-specific raw-richness, rarefied-richness, raw-evenness, probability of interspecific encounter (PIE, Eq. 3, mathematically equivalent to the concept of rarefied-evenness), and number of records of Symbiodiniaceae assemblages associated with 123 coral species. Coral host species-specific bathymetric, latitudinal, and longitudinal ranges and bleaching response are included.

**Additional file 3: Table S3.** Phylogenetic signal evaluation (Pagel’s λ) and correlation coefficients (r) of phylogenetically-corrected and uncorrected linear correlations throughout the study. Significant Pagel’s λ *p*-values (< 0.05, in bold) indicate phylogenetic bias in the distribution of linear regression residuals; and only when Pagel’s λ is significant, were phylogenetically-corrected regression results accepted for interpretation. The *p*-values of regressions indicated by Pagel’s λ test (either linear for non-significant λ, or phylogenetic for significant λ) are also in bold to indicate which significant result was accepted for interpretation.

**Additional file 4: Figure S1.** Key to individual species rarefaction curves shown in Fig. 2a.

**Additional file 5: Figure S2.** Frequency of coral-Symbiodiniaceae records of association (out of a total of 15,556 records) identified with different ITS2 primer sets described in the reports included in the dataset. See Table S1 for details of individual studies and their specific protocols and primer sets.

## Data Availability

All data generated or analyzed during this study are included in this published article and its supplementary information files.

## Abbreviations

BRI: Bleaching response index
*cob*: Cytochrome b
DGGE: Denaturing gradient gel electrophoresis
*ITS*: Internal transcribed spacer
NGS: Next-generation sequencing
OLS: Ordinary least squares
PCR: Polymerase chain reaction
PDAP: Phenotypic Diversity Analysis Programs
PIC: Phylogenetic independent contrasts
PIE: Probability of interspecific encounter
P-IGV: Potential intragenomic variants
*psbA*^*ncr*^: Photosystem II P680 reaction center D1 protein non-coding region
raw-E: Raw evenness
raw-R: Raw richness
rDNA: Ribosomal deoxyribose nucleic acid
RNA: Ribose nucleic acid
