# Supplementary figures and images for: Uncovering the role of Symbiodiniaceae assemblage composition and abundance in coral bleaching response by minimizing sampling and evolutionary biases
Source: BMC Microbiol. 2020 May 19;20:124. doi: 10.1186/s12866-020-01765-z (PMC7236918; doi:10.1186/s12866-020-01765-z)

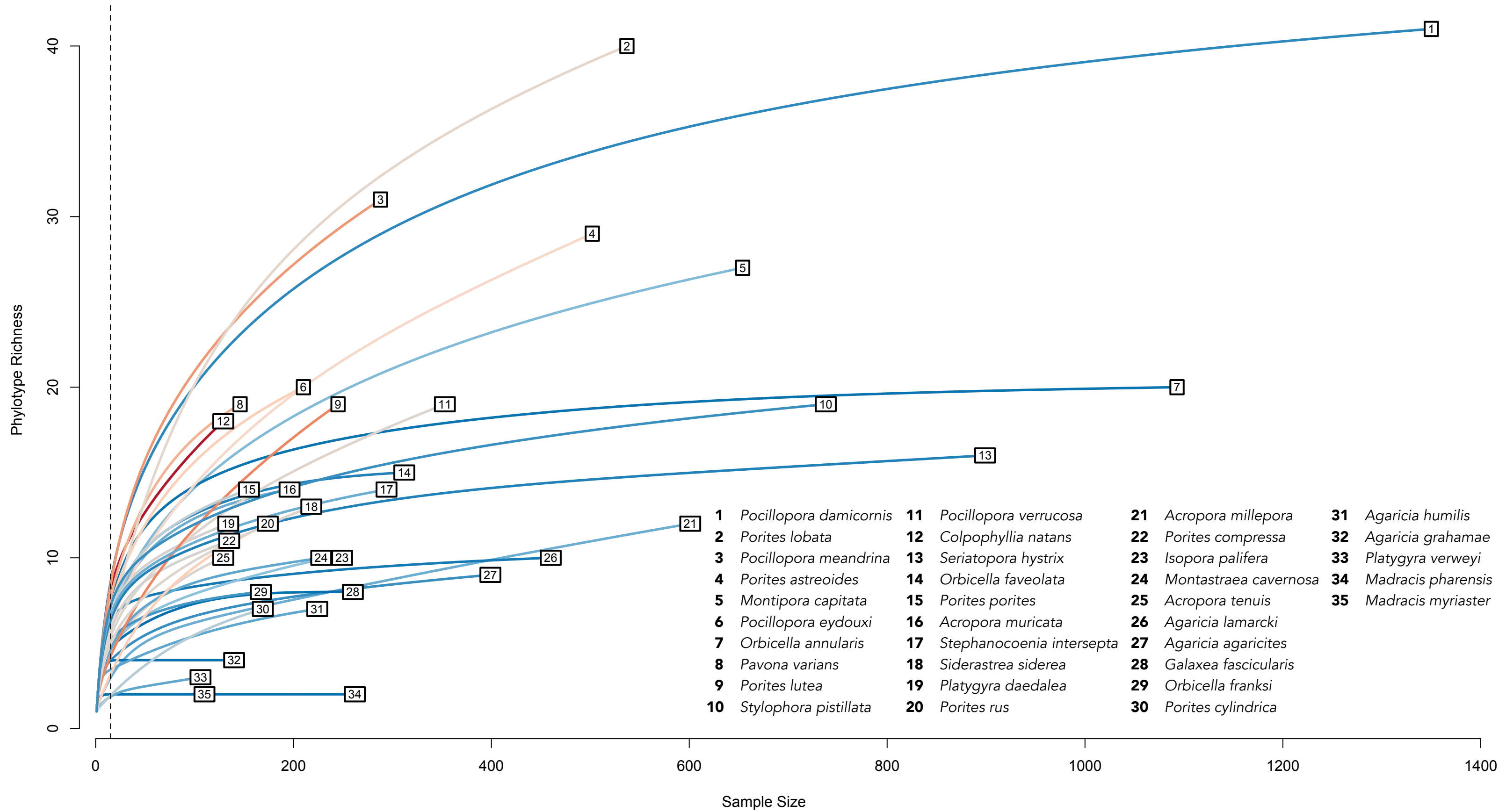

Supplement: Supplementary file 4 — Additional file 4: Figure S1. Key to individual species rarefaction curves shown in Fig. 2a. [file 12866_2020_1765_MOESM4_ESM.pdf]

ITS Primers used in this study (15,566 records)

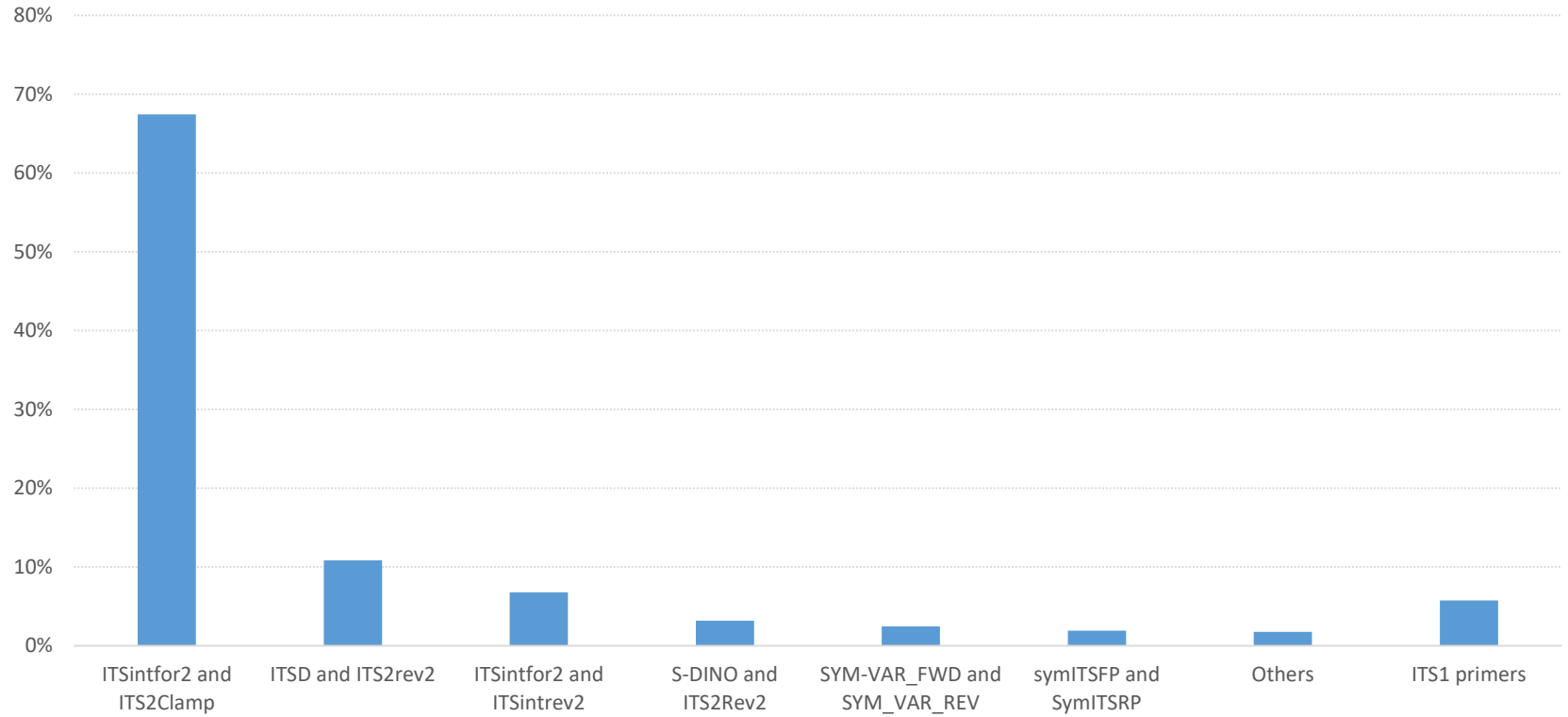

Supplement: Supplementary file 5 — Additional file 5: Figure S2. Frequency of coral-Symbiodiniaceae records of association (out of a total of 15,556 records) identified with different ITS2 primer sets described in the reports included in the dataset. See Table S1 for details of individual studies and their specific protocols and primer sets. [file 12866_2020_1765_MOESM5_ESM.pdf]
